# Serum Uric Acid and the Risk of Dementia: A Systematic Review and Meta-Analysis

**DOI:** 10.3389/fnagi.2021.625690

**Published:** 2021-02-25

**Authors:** Zhike Zhou, Shanshan Zhong, Yifan Liang, Xiaoqian Zhang, Rongwei Zhang, Kexin Kang, Huiling Qu, Ying Xu, Chuansheng Zhao, Mei Zhao

**Affiliations:** ^1^Department of Geriatrics, The First Affiliated Hospital, China Medical University, Shenyang, China; ^2^Department of Neurology, The First Affiliated Hospital, China Medical University, Shenyang, China; ^3^Department of Neurology, People's Hospital of Liaoning Province, Shenyang, China; ^4^Computational Systems Biology Laboratory, Department of Biochemistry and Molecular Biology and Institute of Bioinformatics, The University of Georgia, Athens, GA, United States; ^5^Cancer Systems Biology Center, The China–Japan Union Hospital, Jilin University, Changchun, China; ^6^Department of Cardiology, The Shengjing Affiliated Hospital, China Medical University, Shenyang, China

**Keywords:** uric acid, dementia, Alzheimer's disease, risk factor, meta-analysis

## Abstract

**Background:** This meta-analysis aimed to evaluate the relationship between serum uric acid (UA) and the risk of dementia and its subtypes.

**Methods:** Embase, PubMed, and Web of Science were searched from inception to July 2020. Random-effect models were employed to analyze the standard mean difference (SMD) with the corresponding 95% confidence intervals (CI).

**Results:** Twenty-three eligible studies involving 5,575 participants were identified. The overall results showed lower levels of UA in dementia relative to non-dementia controls [SMD = −0.32 (−0.64; −0.01) *p* = 0.04]. The subgroup analysis of the type of dementia demonstrated a significant association of UA with Alzheimer's disease (AD) [SMD = −0.58 (−1.02; −0.15) *p* = 0.009] and Parkinson's disease with dementia (PDD) [SMD = −0.33 (−0.52; −0.14) *p* = 0.001] but not with vascular dementia (VaD). The stratification analysis of the concentrations of UA revealed that the UA quartile 1–2 was negatively correlated with dementia and neurodegenerative subtypes (*p* < 0.05), whereas a positive correlation of UA quartile 4 with dementia was noted (*p* = 0.028). Additionally, the meta-regression analysis on confounders showed that not age, body mass index, diabetes mellitus, hypertension, or smoking but education (*p* = 0.003) exerted an influence of the UA in the risk estimate of dementia.

**Conclusions:** Low concentrations of UA (< 292 μmol/L or 4.91 mg/dL) is a potential risk factor for AD and PDD but not for VaD. The mechanism of different concentrations of the UA in dementia needs to be confirmed through further investigation.

## Introduction

Dementia is a group of acquired clinical syndromes characterized by the progressive decline in cognition along with psychiatric and behavioral alterations of differing extents (Tari et al., 2019; Zhou et al., 2020). More than 50 million people worldwide suffer from the overload burden of finance due to this clinical entity, a number that is alarmingly expected to triple by 2050 (Kivipelto et al., 2020). The seriousness and urgency of the present situation are that no licensed drugs are available to cure any type of dementia, such as Alzheimer's disease (AD), Parkinson's disease with dementia (PDD), and vascular dementia (VaD) (Jennings et al., 2020). Incurable, however, does not mean untreatable. Approximately one-third of the cases of AD are partially due to modifiable risk factors, many of which are related to lifestyles (e.g., smoking, physical inactivity, midlife obesity, and hypertension) (Norton et al., 2014). Subsequently, attention to lifestyle interventions has increasingly concentrated on patients with diagnostic dementia and the general population at a high risk of developing dementia (Ngandu et al., 2015).

Serum uric acid (UA), regulated by diet and physical activities, is the final breakdown of purine nucleotides that plays a crucial role in human metabolism (So and Thorens, 2010; Viazzi et al., 2016). Observational studies have shown that hyperuricemia was a determinant of long-term hypertension (Bjornstad et al., 2015), possibly representing an association of UA with renal and cardiovascular diseases (Feig et al., 2008; Kanbay et al., 2013). Despite extensive estimates of the risk of dementia based on UA, the conclusion remained ambiguous. Several lines of evidence suggested UA as an index of redox homeostasis, which alleviated the presence of degenerative cascades in neurodegenerative diseases (e.g., AD, PD, frontotemporal dementia, progressive supranuclear palsy, and corticobasal degeneration) (Vannorsdall et al., 2008; Paganoni and Schwarzschild, 2017; Schirinzi et al., 2017). On the other hand, serum UA might aggravate cerebral ischemia and increase the risk of dementia (Vannorsdall et al., 2008). To address these conflicts, an integrated retrieve of literature was undertaken to summarize the population-based data on the correlation between UA and dementia, aiming to afford a quantitative assessment for the risk of dementia and its subtypes under different concentration of UA.

## Materials and Methods

### Inclusion and Exclusion Criteria

Published studies that met the following criteria were considered eligible: (1) determinant dementia in cases (such as AD, PDD, and VaD) had to be based on certain standardized diagnostic criteria, e.g., the National Institute of Neurological and Communicative Disorders and Stroke–Alzheimer's Disease and Related Disorders Association (NINCDS–ADRDA) (McKhann et al., 1984), the International Classification of Diseases (ICD)-10 criteria, and the Diagnostic and Statistical Manual of Mental Disorders (DSM)-III, -IV, or -V criteria (American Psychiatric Association, 1980, 1987, 1994, 2013), etc.; (2) participants with no dementia served as controls (including but not limited to healthy individuals and PD with non-dementia); (3) the concentration of serum UA in dementia and matched controls was available in each study; and (4) there were no restrictions of the study design (case-control, longitudinal, or mix of them). The exclusion criteria were as follows: (1) duplicate data or population from the same research center; (2) receiving UA therapy or anti-gout drugs; (3) subjects examined with renal failure dialysis, severe liver dysfunctions, or malignant diseases; (4) non-English publications; and (5) animal studies, case reports, letters, reviews, or conference abstracts.

### Literature Search

Two reviewers retrieved the electronic databases of Embase (August 1973–June 2020), PubMed (September 1974–July 2020), and Web of Science (March 1992–July 2020), which followed the developed guidelines of the preferred reporting items for systematic reviews and meta-analyses (PRISMA) (Moher et al., 2009) (Supplementary Table 1). Search terms in the aforementioned databases were “uric acid”, “urate”, “hyperuricemia”, “dementia”, “cognitive”, “amentia”, and “Alzheimer”. The reference lists of included studies and relevant overviews were studied manually to minimize the omission of potentially eligible articles.

### Data Extraction and Synthesis

The following information of selected studies was independently extracted by two investigators: first author, year, country, detection method, gender distribution, mean age, and levels of UA (Table 1). For discrepancies of the data, we negotiated with a third reviewer or consulted the authors of the original report. Other general characteristics, including body mass index (BMI), education, male gender, smoking, hypertension, and diabetes mellitus, were also summarized. As shown in Table 2, the association between general characteristics and the risk of dementia was evaluated.

**Table 1:** General characteristics of population-based studies on serum UA and dementia.

**First author**	**Year**	**Country**	**Detecting methods**	**Dementia**			**Controls**		
				**Male/n**	**UA, μmol/L**	**Age, Years**	**Male/n**	**UA, μmol/L**	**Age, Years**
Al-khateeb E	2015	Jordan	Colorimetric	25/40	300.48 ± 76.16	71.5 ± 9.11	25/41	343.91 ± 101.15	68.46 ± 8.13
Annanmaki T	2011	Finland	NR	17/28	285 ± 63.8	63.6 ± 15.6	4/12	309.1 ± 87.5	63.40 ± 9.63
Baldeiras I	2008	Portugal	Colorimetric	21/42	230 ± 20	69.9 ± 1.3	10/37	270 ± 20	68.4 ± 1.8
Boccardi V	2020	Italy	Colorimetric	16/56	287.98 ± 77.35	81.26 ± 4.58	35/65	346.29 ± 104.72	77.80 ± 6.09
Cankurtaran M	2013	Turkey	NR	51/143	303.45 ± 85.09	73.52 ± 6.25	588/1553	327.85 ± 88.66	72.45 ± 5.85
Cascalheira JF	2009	Portugal	Colorimetric	10/19	315.35 ± 74.9	75.6 ± 5.8	18/36	261.8 ± 26.4	70.7 ± 7.3
Cervellati C_AD	2014	Italy	ELISA	23/89	344 ± 10.5	78.8 ± 0.8	15/48	317 ± 17.1	77.8 ± 0.7
Cervellati C_VaD	2014	Italy	ELISA	24/54	310 ± 96.3	76.2 ± 7.3	672/1724	312.4 ± 73.4	64.1 ± 5.7
Foy CJ_AD	1999	UK	Colorimetric	49/79	317.4 ± 78.9	76.2 ± 7.3	672/1724	312.4 ± 73.4	64.1 ± 5.7
Foy CJ_PDD	1999	UK	Colorimetric	10/18	260 ± 88.89	72 ± 12.59	32/58	300 ± 103.7	74 ± 8.15
Foy CJ_VaD	1999	UK	Colorimetric	19/37	240 ± 74.07	79 ± 11.85	32/58	300 ± 103.7	74 ± 8.15
González-Aramburu I	2014	Spain	ELISA	35/72	299.88 ± 101.15	79.2 ± 8.5	160/271	318.33 ± 83.3	71.2 ± 11.5
Hatanaka H_AD	2015	Japan	Colorimetric	22/72	298.10 ± 75.57	79.4 ± 6.6	24/53	338.56 ± 99.96	83.2 ± 8.9
Hatanaka H_VaD	2015	Japan	Colorimetric	14/27	323.09 ± 74.38	82.1 ± 7.6	24/53	338.56 ± 99.96	83.2 ± 8.9
Kim TS	2006	Korea	Colorimetric	41/101	261.8 ± 71.4	73.5 ± 8.4	45/101	303.45 ± 65.45	73.2 ± 3
Maesaka JK_AD	1993	America	Colorimetric	NR/18	270 ± 20	79.2 ± 1.8	0/11	350 ± 30	76.7 ± 1.6
Maesaka JK_VaD	1993	America	Colorimetric	NR/6	330 ± 20	80.2 ± 2.2	0/11	350 ± 30	76.7 ± 1.6
Pellecchia MT	2016	Italy	ELISA	14/23	273.7 ± 59.5	62 ± 7.1	11/19	315.35 ± 101.15	56.2 ± 8.2
Polidori MC	2002	Italy	HPLC	0/35	225.1 ± 41	85.9 ± 5.5	0/40	278.3 ± 59.9	85.4 ± 4.4
Pu Z	2017	China	ELISA	30/55	225.71 ± 41.84	77.83 ± 6.47	21/40	351.43 ± 54.68	74.17 ± 6.54
Pulido R	2005	Spain	ELISA	9/10	242 ± 70	69 ± 4	10/22	285 ± 98	61 ± 10
Rinaldi P	2003	Italy	HPLC	17/63	199 ± 51.9	76.8 ± 6.9	20/56	312.9 ± 82.3	75.8 ± 7.2
Serdarevic N	2020	Yugoslavia	ELISA	100/100	321.25 ± 85.75	73.74 ± 8.15	100/100	263 ± 62.5	69.74 ± 7.41
Tohgi H_AD	1993	Japan	NR	NR/10	262 ± 88	68 ± 8	NR/14	303 ± 70	68 ± 6
Tohgi H_VaD	1993	Japan	NR	NR/15	283 ± 91	69 ± 6	NR/14	303 ± 70	68 ± 6
Tuven B_PDD	2017	Turkey	Colorimetric	NR/15	277.27 ± 111.86	NR	NR/1119	343.91 ± 108.29	NR
Tuven B_VaD	2017	Turkey	Colorimetric	NR/16	376.04 ± 181.48	NR	NR/1119	343.91 ± 108.29	NR
Wang C	2018	China	NR	38/90	294.23 ± 84.7	77.61 ± 7.2	38/90	333.39 ± 96.44	77.36 ± 6.74
Xu Y	2016	China	Colorimetric	69/127	300.12 ± 110.48	67.4 ± 7.8	43/81	336.59 ± 103.63	68.1 ± 8.2
Zuliani G	2018	Italy	ELISA	23/90	357 ± 95	77 ± 6	9/81	320 ± 96	69 ± 9

*AD, Alzheimer's disease; ELISA, enzyme-linked immunosorbent assay; HPLC, high-performance liquid chromatography; n, number; NR, not reported; PDD, Parkinson's disease with dementia; VaD, vascular dementia; UA, uric acid*.

**Table 2:** Pooled weighted characteristics.

	**Dementia vs. control arm**		
	**SMD**	**95% CI**	***P*-value**
Age	0.51	(0.30, 0.71)	0.001
Body mass index	−0.07	(−0.23, 0.10)	0.429
Education	−1.69	(−2.78, −0.60)	0.002
	**Odds ratio**	**95% CI**	***P*****-Value**
Male gender	1.01	(0.80, 1.27)	0.933
Smoking	1.08	(0.72, 1.62)	0.698
Hypertension	1.32	(0.60, 2.91)	0.490
Diabetes mellitus	1.05	(0.69, 1.60)	0.806

*SMD, standard mean difference; CI, confidence interval*.

### Statistical Analysis

Software STATA version 15.0 and Review Manager 5.3 were used to conduct the meta-analyses. The effect size (ES) with a corresponding 95% confidence interval (CI) was identified by a *z*-test in which continuous and classified variables were measured by standard mean differences (SMD) and odds ratio (OR), respectively. The statistical heterogeneity between studies was assessed by the *I*^2^ test, with a value higher than 50% considering heterogeneity (Zintzaras and Ioannidis, 2005). A random-effect model was appropriate if there was substantial heterogeneity within the study populations (DerSimonian and Laird, 1986). Subgroup analyses on the type of dementia (AD, VaD, and PDD) and the concentrations of serum UA (quartile 1–4: < 262, 262–292, 292–316, > 316 μmol/L; or < 4.40, 4.40–4.91, 4.91–5.31, > 5.31 mg/dL) were carried out in levels of UA for the risk estimates of dementia. Meta-regression analyses were then applied to investigate how potential confounding factors affected the correlation of UA to dementia. A sensitivity analysis was conducted by omitting each study to assess its impact on the pooled ES. For continuous data with the mean difference as the effect index, Egger's linear regression test was selected, and *p* ≥ 0.05 suggested a low possibility of publication bias (Peters et al., 2006).

## Results

### Study Selection and Characteristics

**Figure 1:**
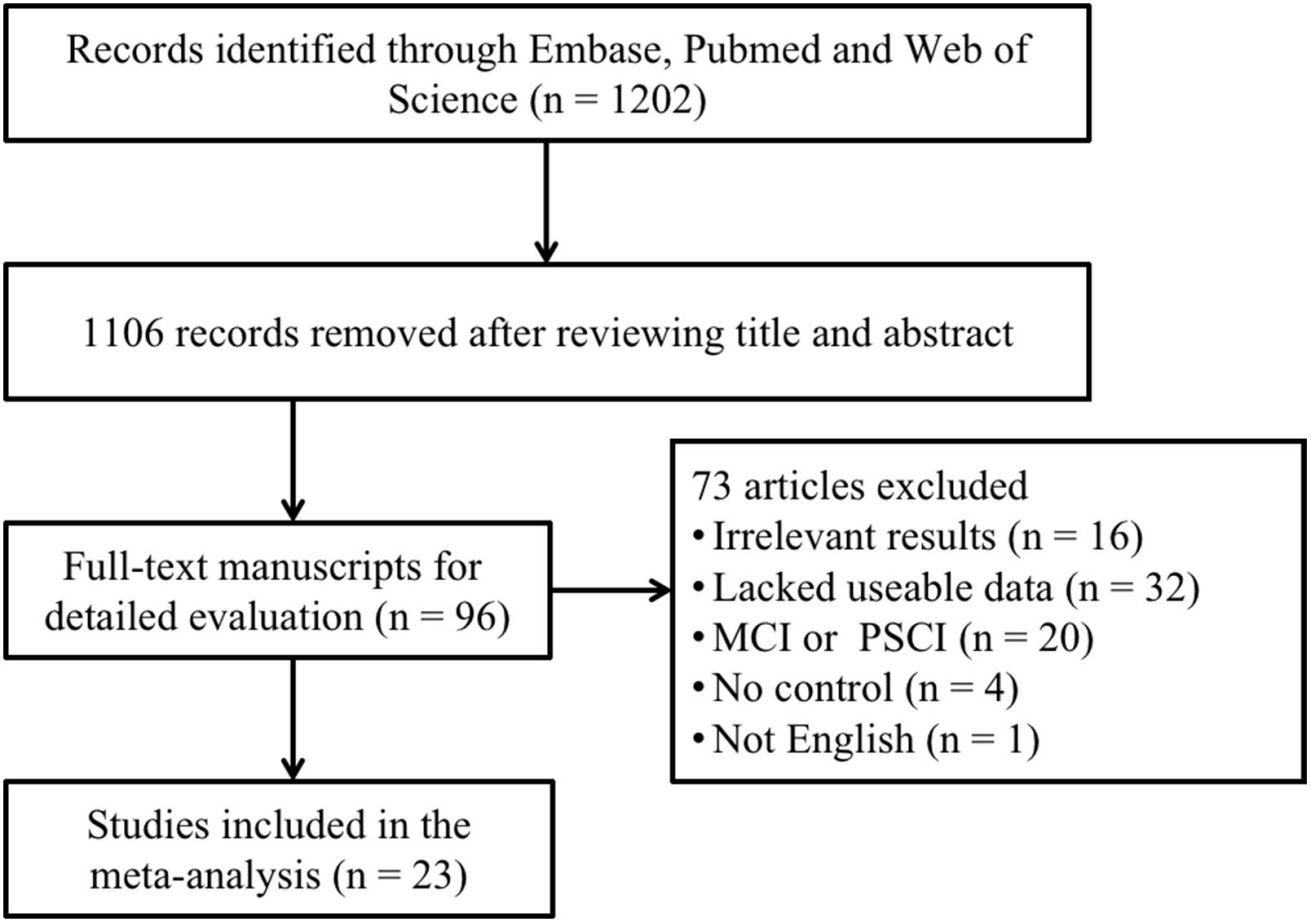
A flowchart of the screening of the study and the selection in the meta-analysis. MCI, mild cognitive impairment; PSCI, post-stroke cognitive impairment.

**Figure 2:**
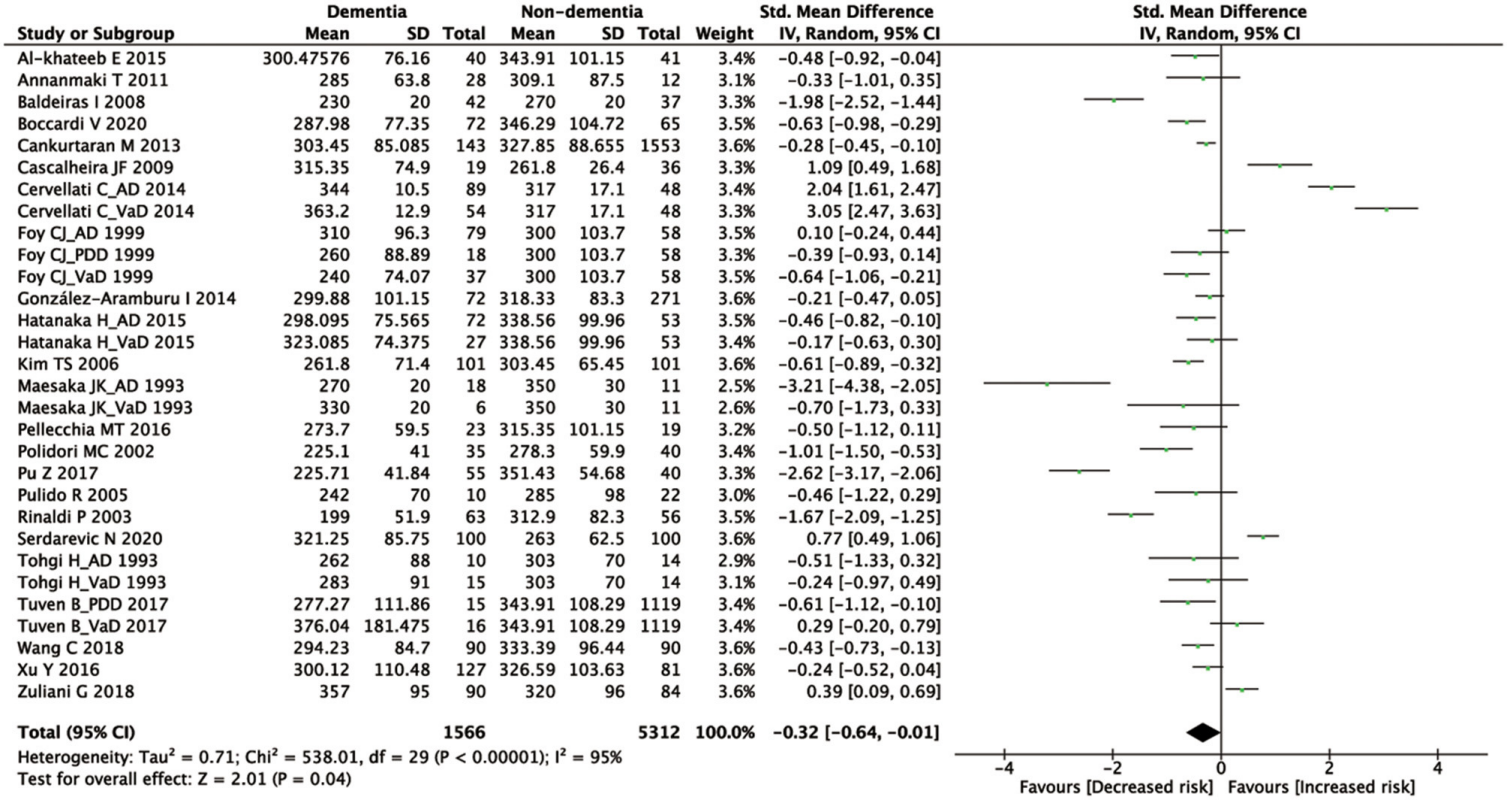
A forest plot of the SMD in the levels of UA between dementia and non-dementia. CI, confidence interval; SMD, standard mean difference; UA, uric acid.

**Figure 3:**
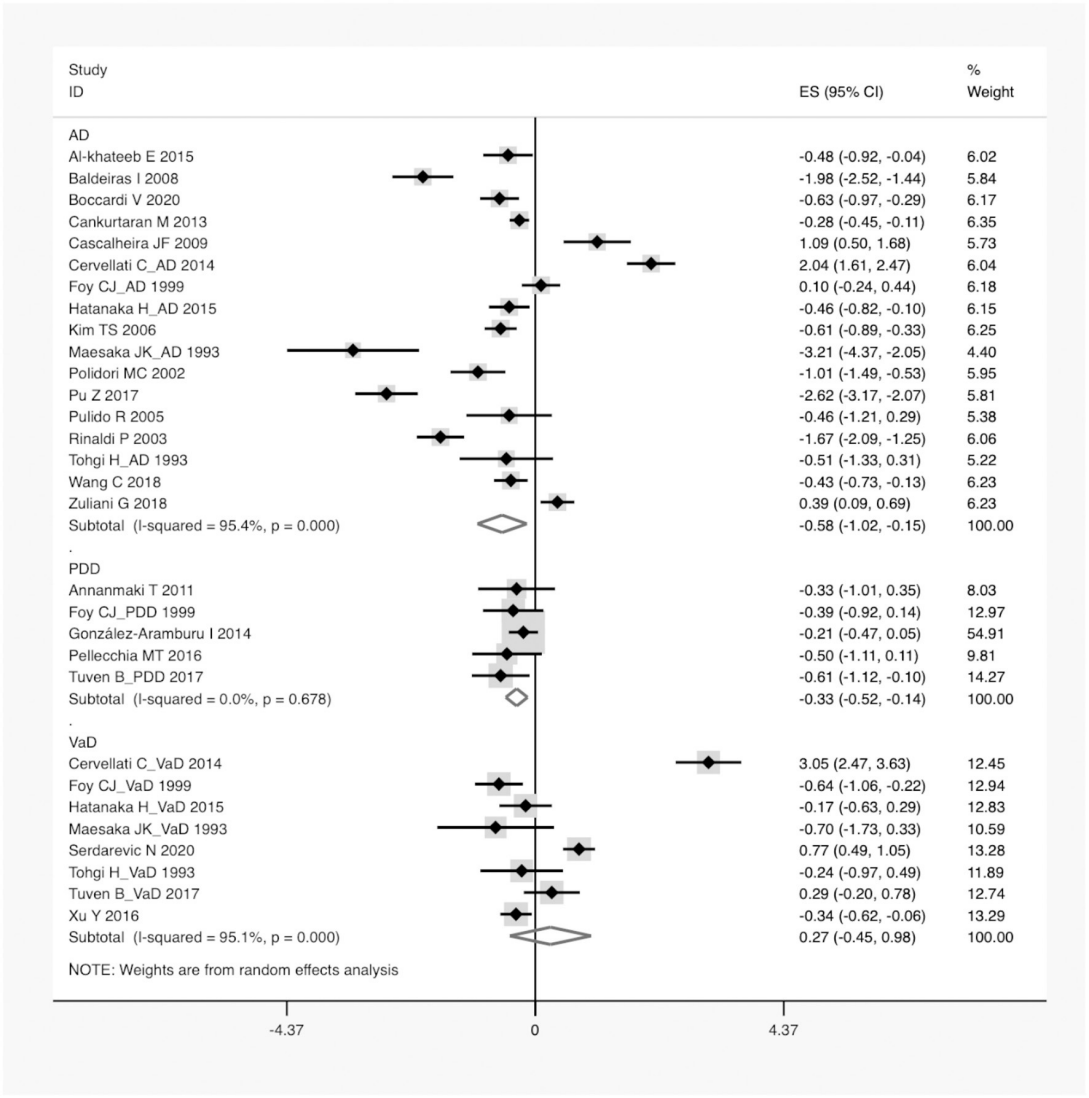
A forest plot of the subgroup analysis on the type of dementia in UA between dementia and non-dementia. AD, Alzheimer's disease; PDD, Parkinson's disease with dementia; VaD, vascular dementia; ES, effect size; CI, confidence interval; UA, uric acid.

**Figure 4:**
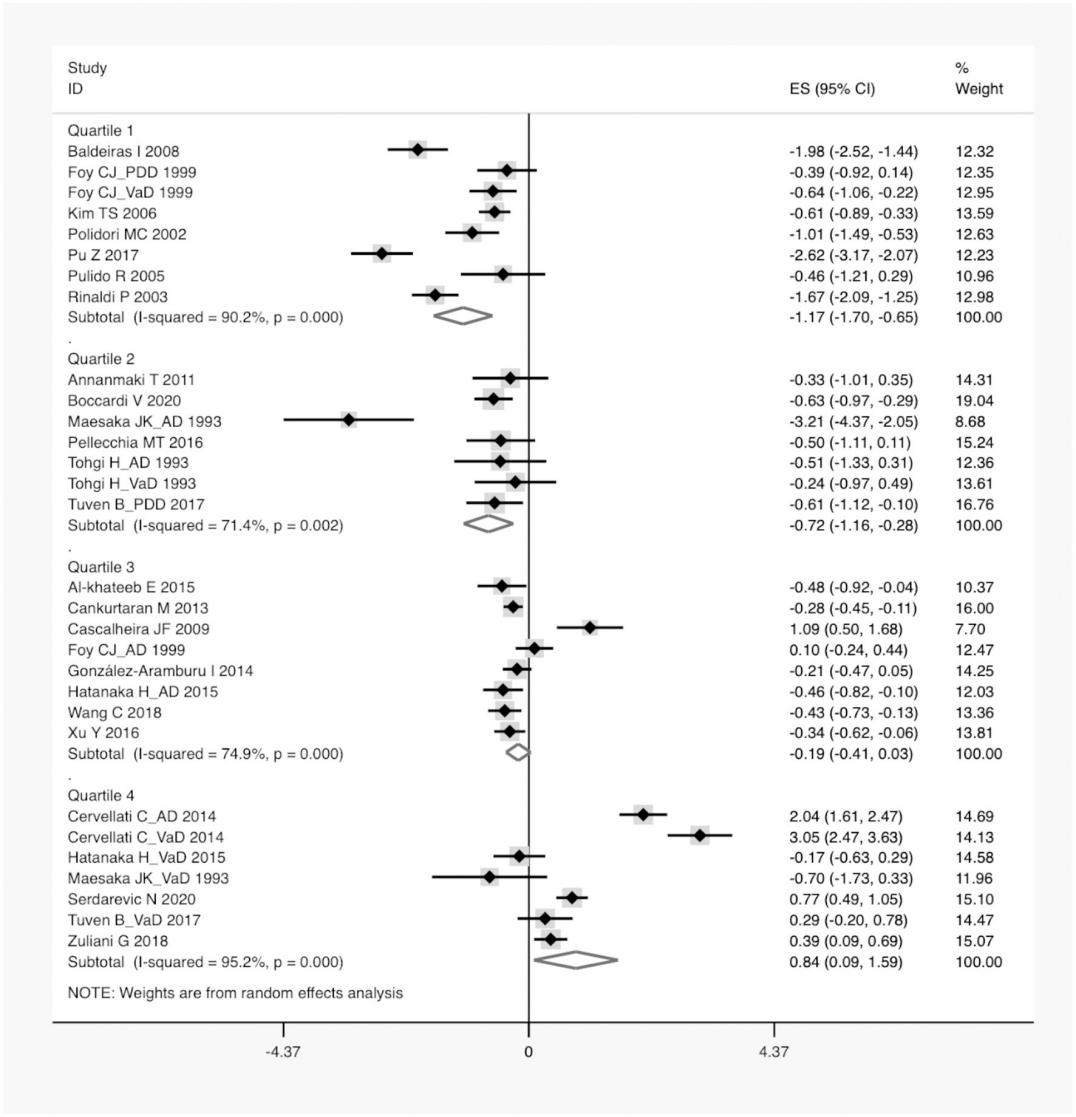
A forest plot of the subgroup analysis on the concentration of serum UA between dementia and non-dementia. ES, effect size; CI, confidence interval; UA, uric acid.

**Figure 5:**
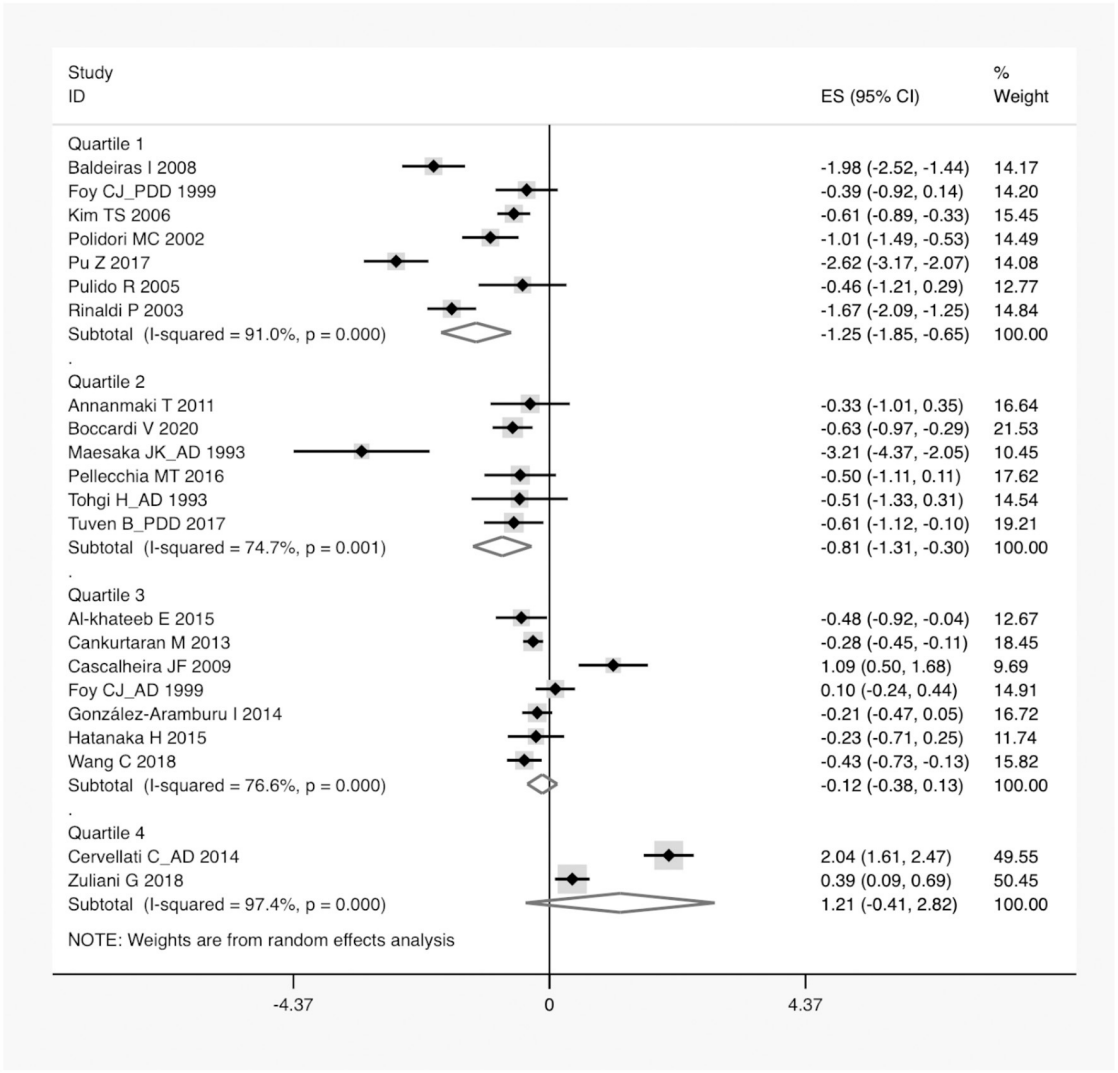
A forest plot of the subgroup analysis on the concentration of serum UA in neurodegenerative dementia compared with non-dementia. ES, effect size; CI, confidence interval; UA, uric acid.

**Figure 6:**
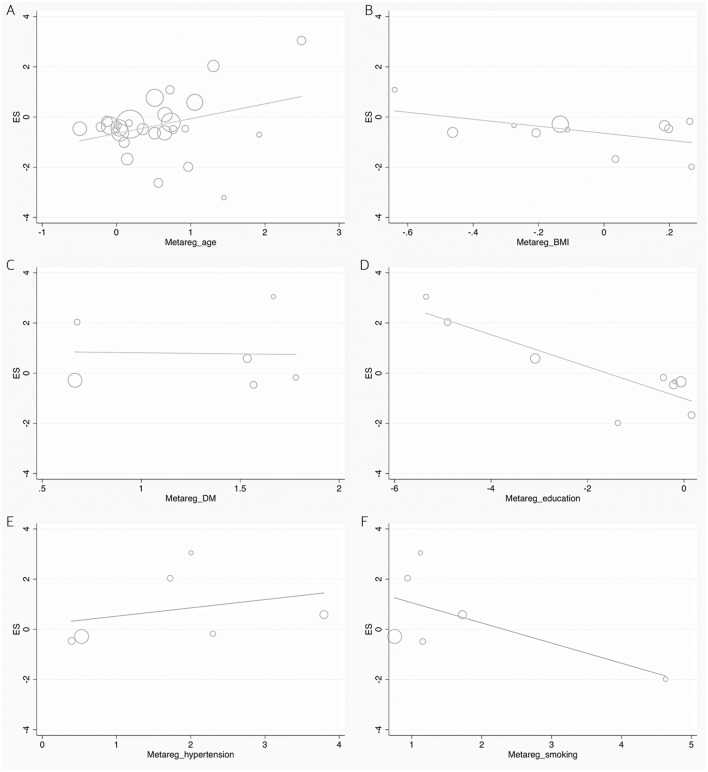
Forest plots of the meta-regression analyses on age **(A)**, BMI **(B)**, DM **(C)**, education **(D)**, hypertension **(E)**, and smoking **(F)** in UA between dementia and non-dementia. BMI, body mass index; DM, diabetes mellitus; ES, effect size; UA, uric acid.

A total of 1,202 articles were screened in the initial search, of which 96 studies were retained by inspecting titles and abstracts. Then, 73 articles were excluded through a comprehensive review of the full texts. Finally, 23 articles, which met the inclusion criteria, were included in the meta-analysis (for detailed steps, see Figure 1) (Maesaka et al., 1993; Tohgi et al., 1993; Foy et al., 1999; Polidori and Mecocci, 2002; Rinaldi et al., 2003; Pulido et al., 2005; Kim et al., 2006; Baldeiras et al., 2008; Cascalheira et al., 2009; Annanmaki et al., 2011; Cankurtaran et al., 2013; Cervellati et al., 2014; González-Aramburu et al., 2014; Al-khateeb et al., 2015; Hatanaka et al., 2015; Pellecchia et al., 2016; Pu et al., 2017; Tuven et al., 2017; Xu et al., 2017; Wang et al., 2018; Zuliani et al., 2018; Boccardi et al., 2020; Serdarevic et al., 2020). Figure 1 outlines a flowchart regarding the screening process of the inclusion of literature. The general characteristics of 23 studies, including 1,566 dementia patients and 4,009 non-dementia controls, are shown in Table 1. The pooled ES of baseline characteristics is summarized in Table 2. The SMD of each study and the overall studies in serum UA for the estimates of dementia risk are exhibited in Figure 2. Subgroup analyses were performed to identify the association between UA and dementia based on the classification of dementia (AD, PDD, and VaD) (Figure 3), concentrations of UA in patients with dementia (Figure 4), and neurodegenerative dementia (AD and PDD) (Figure 5). In the subgroup analysis of PDD, patients with PD without dementia served as the control group. Meta-regression analyses on age (Figure 6A), BMI (Figure 6B), diabetes mellitus (Figure 6C), education (Figure 6D), hypertension (Figure 6E), and smoking (Figure 6F) were conducted to evaluate the impact of these potential confounders on the ES of dementia in relation to UA. Data on physical activities and dietary habits were unavailable for the meta-regression analysis.

### Meta-Analysis

According to the presence of heterogeneity (*I*^2^ = 95%, *p* < 0.01), the random-effect models were used to render the conservation of estimates by the weight disproportionation in small studies. The combined results of 23 studies showed lower levels of UA in patients with dementia compared with patients without dementia [SMD = −0.32 (−0.64; −0.01) *p* = 0.04] (Figure 2). As shown in Table 2, we found no statistic differences of the pooled weighted characteristics on BMI [SMD = −0.07 (−0.23; 0.10) *p* = 0.429], male gender [OR = 1.01 (0.80; 1.27) *p* = 0.933], smoking [OR = 1.08 (0.72; 1.62) *p* = 0.698], hypertension [OR = 1.32 (0.60; 2.91) *p* = 0.490], and diabetes mellitus [OR = 1.05 (0.69; 1.60) *p* = 0.806] between dementia and non-dementia controls; while older age [SMD = 0.51 (0.30; 0.71) *p* = 0.001] and lower educational attainment [SMD = −1.69 (−2.78; −0.60) *p* = 0.002] appeared in patients with dementia than at in controls with non-dementia. The subgroup analysis of the type of dementia (Figure 3) showed an association of the levels of UA with AD [SMD = −0.58 (−1.02; −0.15) *p* = 0.009] and PDD [SMD = −0.33 (−0.52; −0.14) *p* = 0.001] but not with VaD [SMD = 0.27 (−0.45; 0.98) *p* = 0.466]. The UA quartile 1 [SMD = −1.17 (−1.70; −0.65) *p* < 0.01], 2 [SMD = −0.72 (−1.16; −0.28) *p* = 0.02], and 4 [SMD = 0.84 (0.09; 1.59) *p* = 0.028] were significantly related to dementia, whereas no relationship was noted in UA quartile 3 [SMD = −0.19 (−0.41; 0.03) *p* = 0.093] (Figure 4). There was a negative correlation of UA quartile 1 [SMD = −1.25 (−1.85; −0.65) *p* < 0.01] and 2 [SMD = −0.81 (−1.31; −0.30) *p* = 0.02] with neurodegenerative dementia (Figure 5). The meta-regression analyses (Figure 6) revealed an impact of education (*p* = 0.003, Adj R-squared = 72.36%) on the relationship of UA with dementia; but age (*p* = 0.093, Adj R-squared = 10.20%), BMI (*p* = 0.087, Adj R-squared = 21.32%), diabetes mellitus (*p* = 0.952, Adj R-squared = −25.92%), hypertension (*p* = 0.576, Adj R-squared = −15.10%), and smoking (*p* = 0.167, Adj R-squared = 27.46%) exerted no influence on the relationship.

### Sensitivity Analysis and Publication Bias

After removing one of the included studies at a time, a robust result of the pooled ES was presented in the sensitivity analysis (Supplementary Figure 1). The result of the Egger's regression analysis (*p* = 0.997) indicated no evidence of publication bias (Supplementary Figure 2).

## Discussion

In the current meta-analysis, 23 studies, involving 5,575 participants, were generalized to evaluate the relationship between the levels of serum UA and the risk of dementia. In spite of substantial heterogeneity across studies, the pooled ES showed an increased risk of dementia in individuals with lower levels of UA. To the best of our knowledge, this is the first meta-analysis to quantitatively assess the risk of dementia by different gradients of concentrations of UA. Previously, a meta-analysis lacking sufficient statistical power had been conducted under the influence of confounders (Khan et al., 2016). Moreover, it differed from our study in that the post-stroke cognitive impairment (PSCI) and mild cognitive impairment (MCI) were enrolled in VaD and pooled dementia, respectively. Taking into account these limitations, our objective was to perform an updated overview and meta-analysis for the dose-response effects of serum UA in the risk of dementia and its subtypes.

The findings emerging from the subgroup analysis of the type of dementia demonstrated a direct and negative correlation between serum UA and AD, indicating that low levels of UA might be a risk factor of AD. As a major cause of dementia, AD was characterized by amyloid beta (Aβ), neurofibrillary tangles, and neuritic plaques (Ballard et al., 2011). These pathological hallmarks were observed to be associated with the oxidative stress (Desideri et al., 2017). Convincing evidence showed that the oxidative damage to enzymes of glycolysis, tricarboxylic acid cycle, and the biosynthesis of ATP contributed significantly to the pathogenesis of AD and its progression (Butterfield and Halliwell, 2019). UA, a powerful antioxidant and iron chelator, accounted for ~60% of the scavenging capacity of free radicals in the human body (Ames et al., 1981; Davies et al., 1986). In the case of subnormal levels of UA, the resultant accumulation of the oxidative stress enhanced Aβ-activated apoptosis, a process believed to underlie the development of AD (Desideri et al., 2017). By inhibiting the production of the reactive oxygen species, serum UA had neuroprotective implications in brain aging and cognitive impairment, as demonstrated in the Three-City Dijon cohort study (Latourte et al., 2018). In line with our results, two large population-based cohort studies presented a decreased risk of non-VaD in patients with gout (Hong et al., 2015; Lu et al., 2016).

The subgroup analysis of another neurodegenerative dementia, PDD, also exhibited lower concentrations of UA relative to controls with non-dementia. The involvement of low levels of UA has been reported not only in the onset of PD but also in established PD with worse motor symptoms (de Lau et al., 2005; Winquist et al., 2010). It is worth noting that patients with PD with non-dementia served as the control group. The pathological interference of PD on the metabolism of UA tended to be eliminated; thus, to draw the conclusion that low levels of UA were likely an independent causative factor of PDD. This was consistent with additional evidence that elevated levels of UA were a predictor of slow progression in non-motor symptoms of PD, including cognitive impairment (Moccia et al., 2015; Huang et al., 2018). The underlying mechanism of cognitive decline in PD was complex and likely involved several systems (Gratwicke et al., 2015). Among them, a possible interpretation was that the deposition of oxidants and iron in the substantia nigra of PD enhanced the oxidative stress, which impaired the dopaminergic projection from the substantia nigra to the cortex, leading to executive and attentional dysfunctions in PD (Dexter et al., 1989; Fahn and Cohen, 1992). Coincidentally, the antioxidant and iron scavenger traits of UA might provide natural neuroprotection against the dementing process in PD (Schlesinger and Schlesinger, 2008; Annanmaki et al., 2011).

Nevertheless, we found non-significant relationships of UA with VaD, which might account for the reduced ES of the risk of dementia when all subtypes of dementia merged. A plausible explanation was that the vulnerability of ischemic brain to the oxidative stress increased with the decrease in the levels of UA, leading to a series of VaD-related pathological reactions, such as free radical generation, lipid peroxidation, mitochondrial dysfunction, and excitatory toxicity (Sugawara and Chan, 2003; Allen and Bayraktutan, 2009; Amaro et al., 2015). The administration of serum UA has been shown to prevent neurons from excitotoxic insults in acute ischemic stroke, which contributed to the limitation of the infarct growth and an improvement in the behavioral dysfunction (Amaro et al., 2015). Conversely, several lines of evidence presented an interaction of high levels of UA with hypertension and metabolic syndrome, which synergistically aggravated atherosclerosis to yield VaD (Raffaitin et al., 2009; Richette et al., 2014; Borghi et al., 2015). Based on our findings, it was hypothesized that the pro-arteriosclerotic capacity of UA might counteract its antioxidant property, resulting in the ineffectiveness of UA in the development of VaD.

Furthermore, a linear dose-response correlation of serum UA with the risk of dementia was identified in this study. More specifically, lower levels of UA (<292 μmol/L) were negatively correlated with dementia and the subtypes of neurodegerative dementia, whereas a positive association of higher levels of UA (>316 μmol/L) with dementia was found after a smooth transition over a safe interval. This was consistent with previous studies that fluctuations in the concentrations of UA was linked to altered brain function and cognitive decline (Beydoun et al., 2016; Latourte et al., 2018; Tana et al., 2018). In the data of pooled weighted characteristics, the prevalence of dementia increased with an increase in age and lower educational attainment, in line with the epidemiological survey that nearly one in eight individuals suffered from dementia by the age of 80 (Wald et al., 2011). Further meta-regression analyses revealed that not age, BMI, smoking, hypertension, or diabetes mellitus but education exerted an impact on the relationship of UA with the risk of dementia. The sensitivity analysis showed no statistical difference in serum UA after the removal of one study at a time, suggesting the relatively robust ES of pooled results. Large cohort studies on serum UA (not limited to the concentration range in this study) in different types of dementia are required to validate the association between the levels of UA and the risk of dementia.

## Limitations

Several limitations should be noted in our study. Substantial heterogeneity across studies was detected, partially attributed to different attainment of education. Given the inclusion of PD with non-dementia as controls, risk estimates for pooled dementia should be interpreted with caution. Since the individual data of the concentrations of UA were unavailable in each study, there were certain deviations for the subgroup analysis using the mean concentrations of UA. Other potential sources of heterogeneity might be derived from diversities in the cutoff values, detection methods, diagnostic criteria, underlying diseases, dietary habits, and physical activities, some of which have not been analyzed in this meta-analysis and are worthy of further investigation.

## Conclusion

In aggregate, low levels of the concentration of UA (<292 μmol/L or 4.91 mg/dL) were a potential risk factor for AD and PDD rather than VaD. Additional *in vivo* or *in vitro* experiments are needed to elucidate the mechanisms by which changes in the levels of the concentration of UA are underlying causes of dementia.

## Data Availability Statement

The raw data supporting the conclusions of this article will be made available by the authors without undue reservation.

## Author Contributions

The study was conceived by MZ, CZ, and ZZ. The literature search and selection were conducted by SZ and XZ. The data were extracted and analyzed by YL, KK, YX, RZ, and HQ. The rough manuscript was drafted by MZ, CZ, and ZZ. All authors reviewed and approved the final version of the manuscript.

## Conflict of Interest

The authors declare that the research was conducted in the absence of any commercial or financial relationships that could be construed as a potential conflict of interest.

## Supplementary Material

Supplementary Table 1 The developed guidelines of preferred reporting items for systematic reviews and meta-analysis (PRISMA).

Supplementary Figure 1 A forest plot of a sensitivity analysis by omitting the given named study.

Supplementary Figure 2 A funnel plot of the Egger's test to detect the risk of publication bias in the meta-analysis.
