# Supplementary figures and images for: Serum Uric Acid and the Risk of Dementia: A Systematic Review and Meta-Analysis
Source: Front Aging Neurosci. 2021 Feb 25;13:625690. doi: 10.3389/fnagi.2021.625690 (PMC7947796; doi:10.3389/fnagi.2021.625690)

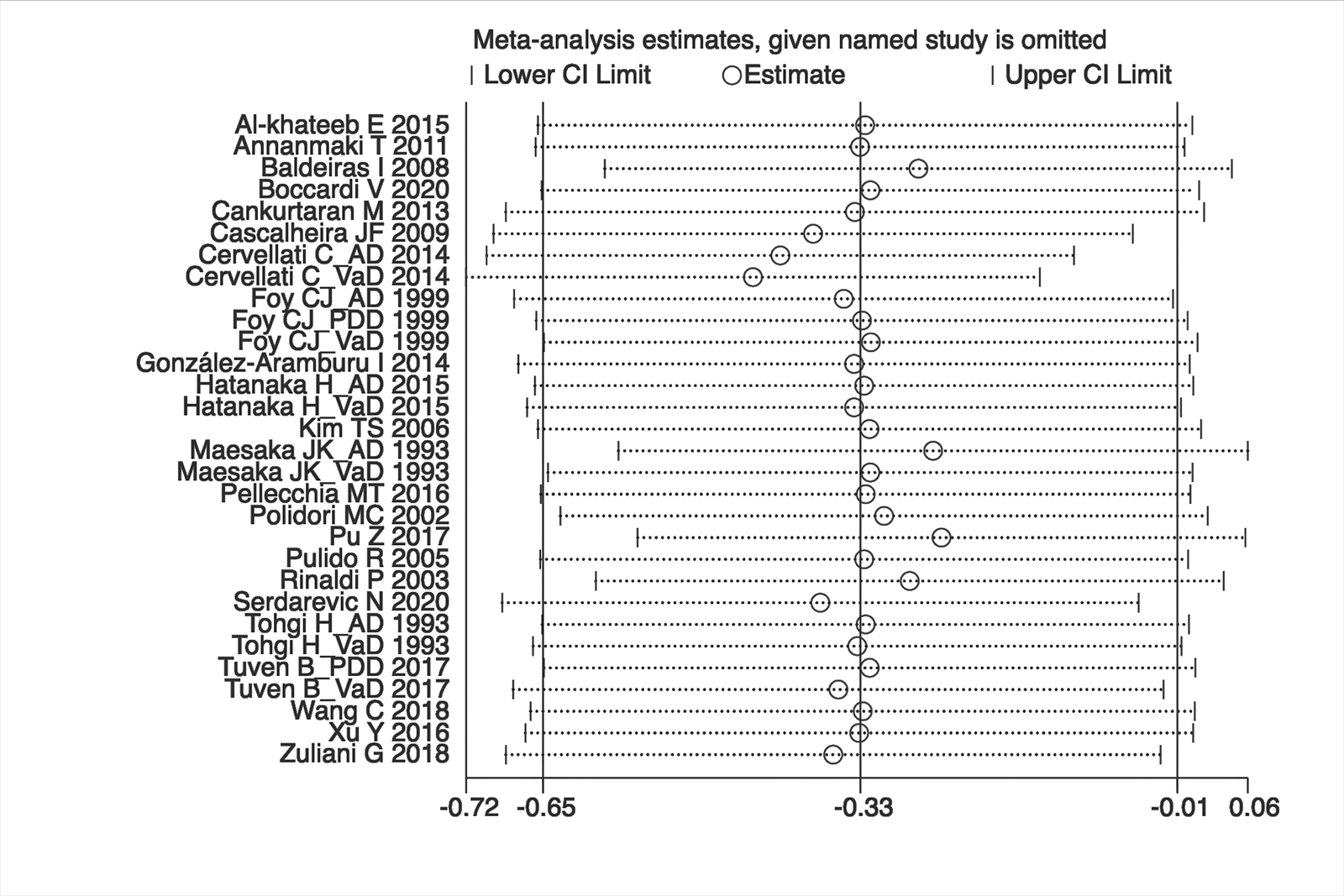

Supplement: Supplementary Figure 1 — A forest plot of a sensitivity analysis by omitting the given named study. [file Image_1.TIF]

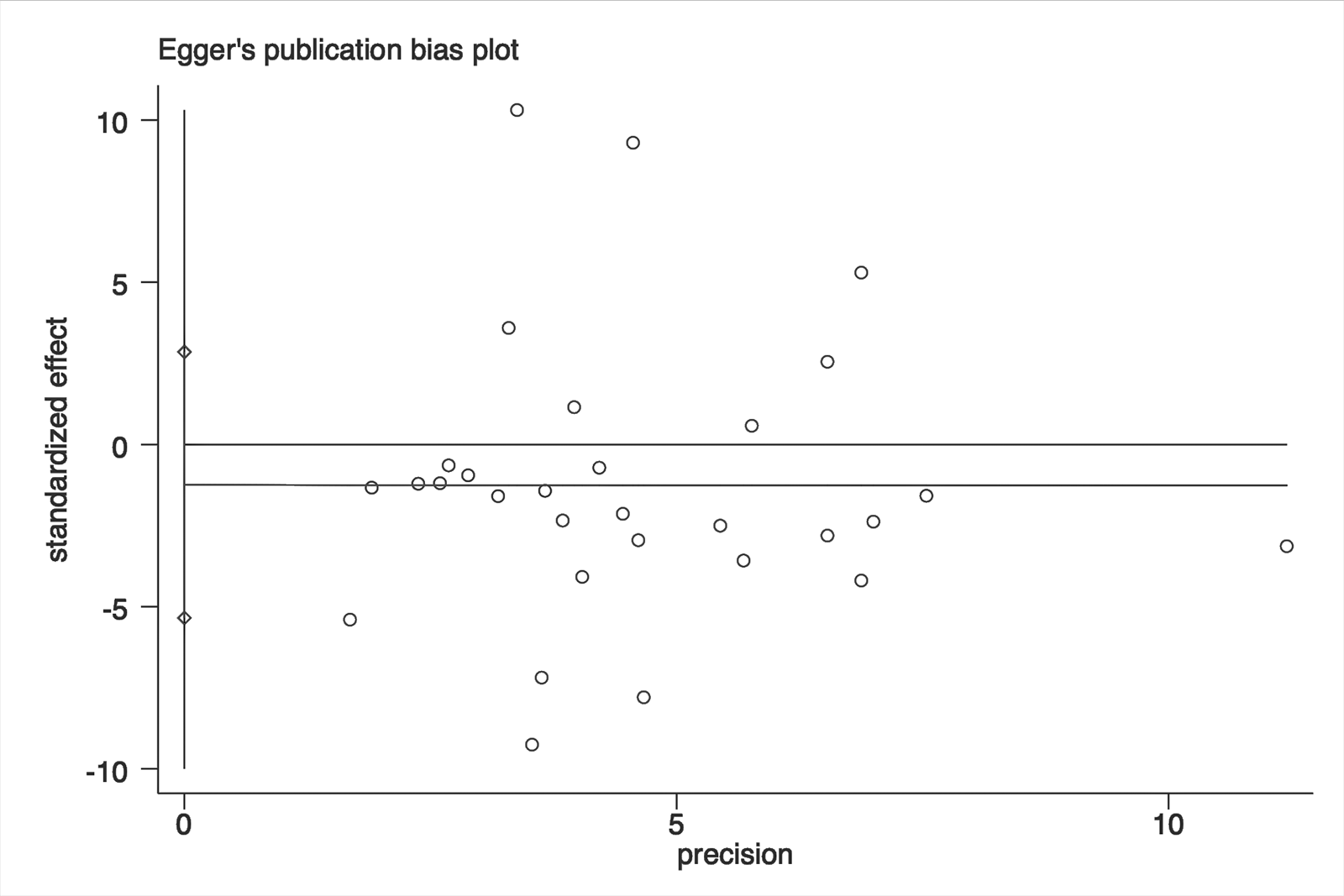

Supplement: Supplementary Figure 2 — A funnel plot of the Egger's test to detect the risk of publication bias in the meta-analysis. [file Image_2.TIF]
